# 1927 nm Thulium Laser Successfully Treats PostInflammatory Hyperpigmentation in Skin of Color

**DOI:** 10.1155/2021/5560386

**Published:** 2021-03-25

**Authors:** Mana Abdullah Alharbi

**Affiliations:** Department of Dermatology, Imam Mohammad Ibn Saud Islamic University, Riyadh, Saudi Arabia

## Abstract

**Background:**

Treatment of postinflammatory hyperpigmentation (PIH) in patients with dark skin is challenging as the treatment itself might provoke paradoxical PIH. Only few studies examined the safety and efficacy of nonablative laser treatment in these patients. The objective was to examine efficacy and safety of nonablative 1927 nm wavelength laser followed by bleaching creams in the treatment of PIH.

**Methods:**

It was a prospective interventional pilot study that was conducted during 2019. All patients were of Fitzpatrick skin type IV who had unsatisfactory response to topical bleaching creams used for at least three months. Patients received one to four sessions of laser treatment (6 weeks apart) followed by topical hydroquinone 4% cream twice daily for 6 weeks. Improvement was assessed by two blinded independent dermatologist evaluators.

**Results:**

A total of nine patients were enrolled and the outcome could not be assessed in one patient who was lost for follow-up. The affected sites were the abdomen, face, and other body parts. Three of the eight evaluated patients had excellent response (37.5%), four had satisfactory response (50.0%), and one had nonsatisfactory response (12.5%). The downtime was manifested as edema and erythema that disappeared after 5 to 7 days. Improvement was more evident in first session and it declined in subsequent sessions. None of the patients had paradoxical pigmentation after treatment.

**Conclusions:**

Low energy low density nonablative fractional 1927 nm wavelength laser treatment followed by topical hydroquinone 4% cream for 6 weeks is a safe and effective modality for improving PIH in patients with darker skin types.

## 1. Introduction

Postinflammatory hyperpigmentation (PIH) is an acquired pigmentary disorder characterized by reactive hypermelanosis of the skin secondary to various endogenous and exogenous conditions [1]. PIH results from the overproduction of melanin or abnormal distribution of melanin pigment deposited in the epidermis and/or dermis [1, 2]. PIH affects all ages and equally affects both genders [3]. It is frequently seen among dark-skinned racial/ethnic groups such as those with African, Asian, and South American ancestry [3]. It represents a common reason for visiting dermatologic clinics in people with darker skin [1, 2]. PIH may develop secondary to several inflammatory dermatoses such as acne, folliculitis, eczema, papulosquamous disorders, and connective tissue diseases [3, 4]. PIH can also develop after skin infections (such as impetigo, chickenpox, and herpes zoster), drug reactions, sunburn, trauma, and friction [3, 4]. Additionally, it occurs following a number of dermatologic procedures such as laser treatment and chemical peeling [3, 4]. The intensity of PIH is probably determined by the inherent skin color and degree and depth of inflammation [4]. The course of the disease is chronic with irregularly shaped lesions that vary in color from light-brown to bluish-grey [2].

The management of PIH is largely dependent on prevention and treatment of the underlying inflammatory conditions [1, 5]. Additionally, topical depigmenting creams, such as hydroquinone, azelaic acid, kojic acid, and arbutin, have been tried with limited success [1, 5]. More recently, a number of nonablative fractional laser treatment modalities have been successfully used in the treatment of PIH [6, 7]. They work by stimulating a robust wound healing after creating zones of microscopic thermal injury surrounded by normal skin to help complete and rapid reepithelization [8, 9]. Treatment of PIH in patients with dark skin is challenging as the treatment itself might provoke inflammatory response and end up exacerbating PIH [10–12]. Additionally, only few studies examined the safety and efficacy of laser treatment in skin of color [13, 14]. The efficacy and safety data of nonablative fractional laser in Saudi patients is limited [15, 16] with no data that focus on new technologies such as 1550 nm/1927 nm dual wavelength laser. The objective of the current study was to examine efficacy and safety of nonablative 1927 nm wavelength laser followed by a depigmenting cream in the treatment of Saudi patients with PIH.

## 2. Methods

### 2.1. Setting and Design

The current study was conducted in a private dermatology practice in Riyadh, Saudi Arabia. It was a prospective interventional pilot study that was conducted during 2019.

### 2.2. Subjects

Patients enrolled in this study were of Fitzpatrick skin type IV who had PIH provoked by different reasons including dermatitis, acne, and chemical peeling as well as previous laser treatments. All included patients had unsatisfactory response to topical bleaching creams used for at least three months before enrolling in this study. Photography was taken before each session and 6 weeks after the last session. Photographs were taken using a digital camera under a constant light setting.

### 2.3. Laser Treatments

Skin preparation was carefully performed with a gentle cleanser to remove debris and makeup before treatment. A topical anesthetic ointment was applied to the treatment area approximately 30 minutes before treatment. The laser treatment used in this study was delivered by Fraxel® DUAL 1550/1927 laser system (Solta Medical, USA). All patients were treated with nonablative 1927 nm thulium fiber laser. Treatments were performed with 30% surface area coverage at pulse energy of 20 mJ (per microthermal zone (MTZ)). Only four passes were done in order to avoid overheating. Patients received one to four sessions (6 weeks apart) according to the response. Patients were given oral steroid 0.5 mg/kg after each session in addition to topical clobetasol cream for 7 days to keep inflammatory response to minimum. Patients were also given topical hydroquinone 4% cream twice daily for 6 weeks starting one week after the laser treatment and strict sunscreen was advised.

### 2.4. Outcome Evaluation

It was done by comparing the before and after digital photographs taken at each visit. Improvement was assessed by two blinded independent dermatologist evaluators using a visual analog scale for the percentage of pigment clearance. The final response was classified as excellent, satisfactory, or nonsatisfactory. Additionally, patients were asked to assess their satisfaction at each visit and during follow-up using a quartile grading scale: grade 1, less than 25% clearance; grade 2, 26–50% clearance; grade 3, 51–75% clearance; grade 4, more than 75% clearance.

### 2.5. Statistical Analysis

Categorical variables were presented as frequencies and percentages. Continuous variables were presented as means and standard deviations (SD). Statistical Package for the Social Sciences software (SPSS Version 25.0; Armonk, NY, IBM Corp) was used for all statistical analyses.

## 3. Results

A total nine patients with Fitzpatrick skin type IV and unsatisfactory response to topical depigmenting creams used for at least three months were enrolled in this study.  Table 1 shows clinical data of the enrolled patients. The affected sites were the abdomen (three patients), face (two patients), forearm, breast, legs, and dorsum of foot (one patient each). The cause of pigmentation was variable and included postprocedure (abdominoplasty, mammoplasty, and liposuction), postlaser treatment (leg and abdomen), eczema, acne, chemical peeling, and burn scare with PIH. The pretreatment duration of pigmentation ranged between 3 and 11 months, with an average of 6.7 ± 2.5 months. The number of laser sessions received ranged between one and four sessions, with an average of 1.7 ± 1.0 sessions. Five patients (55.6%) received one session, three patients (33.3%) received two sessions, and one patient (11.1%) received four sessions.

One patient was lost for follow-up after receiving one session and the downtime and response could not be evaluated. The downtime of the eight patients evaluated ranged between 5 and 7 days, with an average of 5.9 ± 0.8 days. The downtime was manifested as edema and erythema in the first 24 hours followed by superficial crustations at sites of MTZ which slough over later on. According to independent dermatologist evaluation, three of the eight patients had excellent response (37.5%), four patients had satisfactory response (50.0%), and one patient had nonsatisfactory response (12.5%). According to the patient own evaluation, three of the eight patients had grade 4 clearance (37.5%), three had grade 3 clearance (37.5%), and two had grade 2 clearance (25.0%). Low patient satisfaction was observed in postabdominal liposuction and burn scar at the dorsum of foot. Dermatologist and patient evaluations were similar in all but one patient.

**Figure 1:**
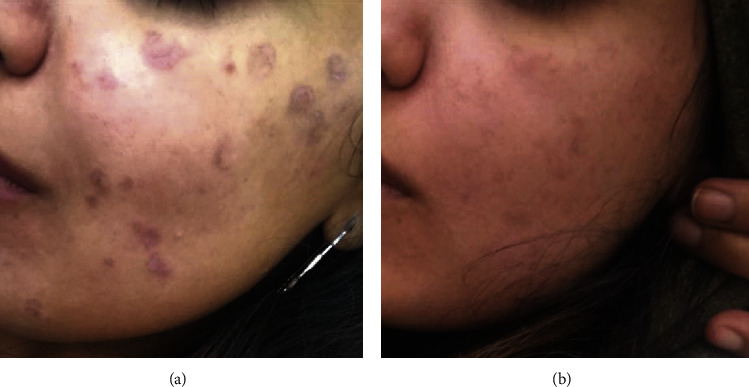
Before and after photographs of a female patient with postinflammatory hyperpigmentation secondary to chemical peeling with excellent response after one session of 1927 nm wavelength laser treatment combined with topical hydroquinone 4% cream for 6 weeks after the laser treatment.

Improvement was more evident in first session and it declined in subsequent sessions. The three patients who had excellent response received only one session while the patient who had nonsatisfactory response received four sessions. Facial lesions had excellent improvement (Figure 1). The pretreatment duration of pigmentation was 5.7 ± 3.0 months in three patients who had excellent response and 7.4 ± 2.5 months in the other five patients. None of the patients had paradoxical pigmentation after treatment.

## 4. Discussion

We are reporting our successful experience in treating patients with PIH of different reasons with nonablative 1927 nm wavelength laser followed by a depigmenting cream. The majority of the current patients had either excellent or satisfactory improvement. Similar findings were reported in the few reports that examined the same wavelength in dark-skinned patients with PIH [13, 14]. For example, Wilson and colleagues reported 50% improvement after 4 laser treatment sessions with or without topical depigmenting cream among 40 patients with dark skin who had facial hyperpigmentation and/or melasma [13]. Similarly, Bae and colleagues reported 43% improvement after at least 2 laser treatment sessions without topical depigmenting cream among 60 patients with dark skin who had PIH [14]. Interestingly, nonablative 1927 nm wavelength laser was reported to have better response in light-skinned patients with PIH [17, 18]. For example, Polder and colleagues reported >75% improvement after three laser treatment sessions among 9 light-skinned patients with nonfacial hyperpigmentation [17]. Similarly, Brauer and colleagues reported marked to very significant improvement in 55% of the 23 patients with largely light skin after 4–6 laser treatment sessions to treat facial PIH or melasma [18].

The excellent or satisfactory improvement in the majority of our patients may be related to the use of topical hydroquinone 4% cream for 6 weeks after the laser treatment. Consistent with this hypothesis, Wilson and colleagues reported a better response to nonablative laser treatment as assessed by Global Aesthetic Improvement Scale at week 12 posttreatment among patients with facial hyperpigmentation who were concomitantly receiving topical hydroquinone compared with those who were concomitantly receiving a bland moisturizer [13]. Additionally, it may be also related to the appropriate choice of the patients with epidermal lesions. For example, nonablative 1927 nm wavelength laser has a higher coefficient for absorption of water compared with the 1,550 nm wavelength emitted by the same DUAL machine, which allows greater ability to target epidermal lesions such as pigmentation and dyschromia [17]. This may also explain the less satisfactory response among the two patients who had PIH with scaring secondary to second degree burn and liposuction. Additionally, scaring may affect the fractional photothermolysis mechanism of laser and consequently obstruct the transepidermal elimination of dermal content [19].

During the current study, we did not encounter any paradoxical pigmentation which might be explained by using low energy low density wavelength in addition to the use of depigmenting cream and strict sun avoidance after treatment. Similarly, no scaring or paradoxical pigmentation was reported in the studies that used nonablative 1927 nm wavelength laser among dark-skinned [13, 14] and light-skinned [17, 20] patients with PIH. The modest downtime experienced by the current patients was similar to what reported before in the form of tolerable pain, moderate erythema, and mild edema that resolve within 7–10 days [17, 20].

Improvement in the current study was more evident in first session and it declined in subsequent sessions. Consistently, Wilson and colleagues showed that the improvement does not considerably change after the first two laser treatment sessions [13]. The improvement was 43.5% after 2 sessions, 44.3% after 3 sessions, 40.6% after 4 sessions, and 43.8% after >5 sessions [13]. Improvement in the current study was better in facial lesions and lesions with shorter pretreatment duration of pigmentation. This might be related to the severity of condition rather than response to laser itself. Lower MTZ density appears to be an important factor to protect against PIH and severe downtime after laser treatment [11, 21, 22].

In conclusion, this pilot study showed that low energy low density nonablative fractional 1927 nm wavelength laser treatment followed by topical hydroquinone 4% cream for 6 weeks after the laser treatment is a safe and effective modality for improving PIH in patients with darker skin types.

## Figures and Tables

**Table 1:** Response to 1927 nm wavelength laser among patients with postinflammatory hyperpigmentation.

	Age	Gender	Affected site	Cause of pigmentation	Skin type	Duration of pigmentation (months)	Number of laser sessions	Downtime (days)	Dermatologist evaluation of improvement	Patient evaluation of improvement	Paradoxical pigmentation
1	22	F	Face	Chemical peeling	IV	3	1	7	Excellent	4	None
2	18	F	Face	Acne	IV	5	1	7	Excellent	4	None
3	28	F	Forearm	Eczema	IV	7	1	5	Satisfactory	3	None
4	39	F	Breast	Postmammoplasty	IV	4	2	6	Satisfactory	3	None
5	25	F	Legs	Laser hair removal	IV	8	2	6	Satisfactory	3	None
6	42	F	Abdomen	Postliposuction	IV	7	4	5	Not satisfactory	2	None
7	21	F	Abdomen	Postcarbondioxide laser	IV	9	1	6	Excellent	4	None
8	30	F	Dorsum of foot	Burn scar with PIH	IV	11	2	5	Satisfactory	2	None
9	44	F	Abdomen	Postabdominoplasty	IV	6	1	NA	NA	NA	NA

## Data Availability

All data used in this study are available upon request.
